# Comparing the effect of a decision aid plus patient navigation with usual care on colorectal cancer screening completion in vulnerable populations: study protocol for a randomized controlled trial

**DOI:** 10.1186/1745-6215-15-275

**Published:** 2014-07-08

**Authors:** Alison T Brenner, Christina M Getrich, Michael Pignone, Robert L Rhyne, Richard M Hoffman, Andrew McWilliams, Brisa Urquieta de Hernandez, Mark A Weaver, Hazel Tapp, Khalil Harbi, Daniel Reuland

**Affiliations:** 1 Cecil Sheps Center for Health Services Research, University of North Carolina at Chapel Hill, 725 Martin Luther King Jr Boulevard, Campus Box 7590, Chapel Hill, NC 27599-7590, USA; 2 Department of Anthropology, University of Maryland, College Park, MD, USA; 3 Division of General Internal Medicine, University of North Carolina School of Medicine, Chapel Hill, NC, USA; 4 Department of Family and Community Medicine, University of New Mexico School of Medicine, Albuquerque, NM, USA; 5 Department of Internal Medicine, University of New Mexico School of Medicine, Albuquerque, NM, USA; 6 University of New Mexico Cancer Center, Albuquerque, NM, USA; 7 Department of Family Medicine, Carolinas HealthCare System, Charlotte, USA; 8 Departments of Medicine and Biostatistics, University of North Carolina at Chapel Hill, Chapel Hill, NC, USA

**Keywords:** colon cancer, colonic neoplasms, decision aids, early detection of cancer, Hispanic Americans, minority health, patient navigation, vulnerable populations

## Abstract

**Background:**

Screening can reduce colorectal cancer (CRC) incidence and mortality. However, screening is underutilized in vulnerable patient populations, particularly among Latinos. Patient-directed decision aids can increase CRC screening knowledge, self-efficacy, and intent; however, their effect on actual screening test completion tends to be modest. This is probably because decision aids do not address some of the patient-specific barriers that prevent successful completion of CRC screening in these populations. These individual barriers might be addressed though patient navigation interventions. This study will test a combined decision aid and patient navigator intervention on screening completion in diverse populations of vulnerable primary care patients.

**Methods/Design:**

We will conduct a multisite, randomized controlled trial with patient-level randomization. Planned enrollment is 300 patients aged 50 to 75 years at average CRC risk presenting for appointments at two primary clinics in North Carolina and New Mexico. Intervention participants will view a video decision aid immediately before the clinic visit. The 14 to 16 minute video presents information about fecal occult blood tests and colonoscopy and will be viewed on a portable computer tablet in English or Spanish. Clinic-based patient navigators are bilingual and bicultural and will provide both face-to-face and telephone-based navigation. Control participants will view an unrelated food safety video and receive usual care. The primary outcome is completion of a CRC screening test at six months. Planned subgroup analyses include examining intervention effectiveness in Latinos, who will be oversampled. Secondarily, the trial will evaluate the intervention effects on knowledge of CRC screening, self-efficacy, intent, and patient-provider communication. The study will also examine whether patient ethnicity, acculturation, language preference, or health insurance status moderate the intervention effect on CRC screening.

**Discussion:**

This pragmatic randomized controlled trial will test a combined decision aid and patient navigator intervention targeting CRC screening completion. Findings from this trial may inform future interventions and implementation policies designed to promote CRC screening in vulnerable patient populations and to reduce screening disparities.

**Clinical trial registration:**

ClinicalTrials.gov NCT02054598.

## Background

### Colorectal cancer screening in vulnerable populations

Colorectal cancer (CRC) is an important cause of cancer death among all men and women in the United States, including Latinos [1,2]. Compared with non-Latinos, Latinos have substantially lower CRC screening rates and may also be more likely to be diagnosed with CRC at an advanced stage [1,3,4]. Despite increases in screening rates in the last decade [5], only 65% of US adults are up to date with recommended screening, and only about 47% of US Latino adults [6,7]. Members of vulnerable groups, including racial or ethnic minorities, the uninsured, and Medicaid populations have the lowest screening rates in the USA [8]. The many patient-, provider-, and system-level barriers that inhibit the CRC screening process disproportionately affect vulnerable groups. Common barriers to completing screening include poor knowledge about screening and screening options, competing demands in primary care, leading to insufficient communication with the provider about screening, lack of health insurance (resulting in a lack of access to care, including cancer screening), and an inability to navigate the healthcare system (resulting in screening not being scheduled or completed). Latinos, now one of the nation’s largest racial or ethnic minority groups, often face additional language (communicating with providers about screening) and cultural barriers (machismo, fatalism) [8-10]. To increase CRC screening in vulnerable populations, interventions that address multiple screening barriers are needed. Moreover, to address ethnic disparities in CRC screening, these interventions must be effective in Latino populations.

### Successful CRC screening requires multiple steps

Successful completion of CRC screening in primary care requires progression through a number of steps: having awareness of and knowledge about screening, deciding that one is ready for screening (intent), having self-efficacy to discuss screening with a provider, and ability to communicate effectively and form a screening plan with a provider. Later steps in the progression include movement from test ordering to test completion. Failures in CRC screening may occur from ‘breakdowns’ of the process at any one of these steps [11,12]. Vulnerable populations are particularly susceptible to such breakdowns, owing to barriers at the levels of the healthcare system and providers, including a lack of access to care and decreased physician time during a visit [13,14]. At the individual level, many factors affect screening uptake, particularly in vulnerable immigrant populations, including: acculturation and language barriers; sociocultural beliefs, such as cancer fatalism; and lack of knowledge and low health literacy [9,15-18].

### Decision aids and patient navigation

Decision aids are evidence-based patient education tools designed to promote informed and shared health-related decision making [19]. Decision aids for CRC screening have the potential to mitigate some of the barriers to screening. For example, decision aids can overcome literacy barriers by having a narrator read all text aloud or by using easy-to-understand graphics and animations. Because they can be viewed outside of the actual patient-provider encounter and can be delivered by other members of the healthcare team, decision aids can also help to overcome provider barriers, such as lack of time to educate patients about screening. Decision aids are designed not only to help patients gain knowledge and build intent, but also to help patients understand their options and prepare them to engage in making informed decisions with their healthcare providers [20]. Studies in English-speaking populations have shown that decision aids can increase patients’ knowledge about cancer and screening, intent to be screened, ability to state a CRC screening test preference, and even screening completion [21-24]. A preliminary study of the Spanish-language decision aid used in this study suggests that the decision aid is efficacious; decision specific knowledge about CRC screening, and self-efficacy and intention to complete CRC screening all improved. It also suggested that the decision aid improved communication between doctor and patient about CRC screening [25]. However, the overall body of evidence is mixed regarding the effect of decision aids as a single intervention on CRC screening test completion. This suggests that while decision aids educate and activate patients, other barriers that inhibit the progression to test completion are not addressed by decision aids alone.

Introduced in the 1990s, patient navigation has been advocated as an approach to addressing barriers to cancer care (including cancer screening) for vulnerable populations [26,27]. Experts currently define patient navigation as a ‘barrier-focused’ intervention that (a) provides support to individual patients for a specific episode of cancer-related care; (b) has a defined endpoint, when an episode of care is complete (for example, CRC screening); (c) targets a defined set of health services required to complete an episode of cancer-related care; and (d) focuses on individual barriers to accessing cancer care [28]. Emerging evidence supports the effectiveness of patient navigation in increasing cancer screening in general and, specifically, in increasing CRC screening in vulnerable populations [29,30]. Decision aids and patient navigators represent potentially complementary interventions for promoting screening because they address multiple barriers that affect different steps in the screening process. Combing the two intervention methods might be a more effective way to address the complex, multilevel barriers to CRC screening that vulnerable populations encounter. However, to our knowledge, no study has tested an intervention combining a decision aid with patient navigation to promote CRC screening in vulnerable populations in a primary care setting.

### Conceptual framework

**Figure 1:**
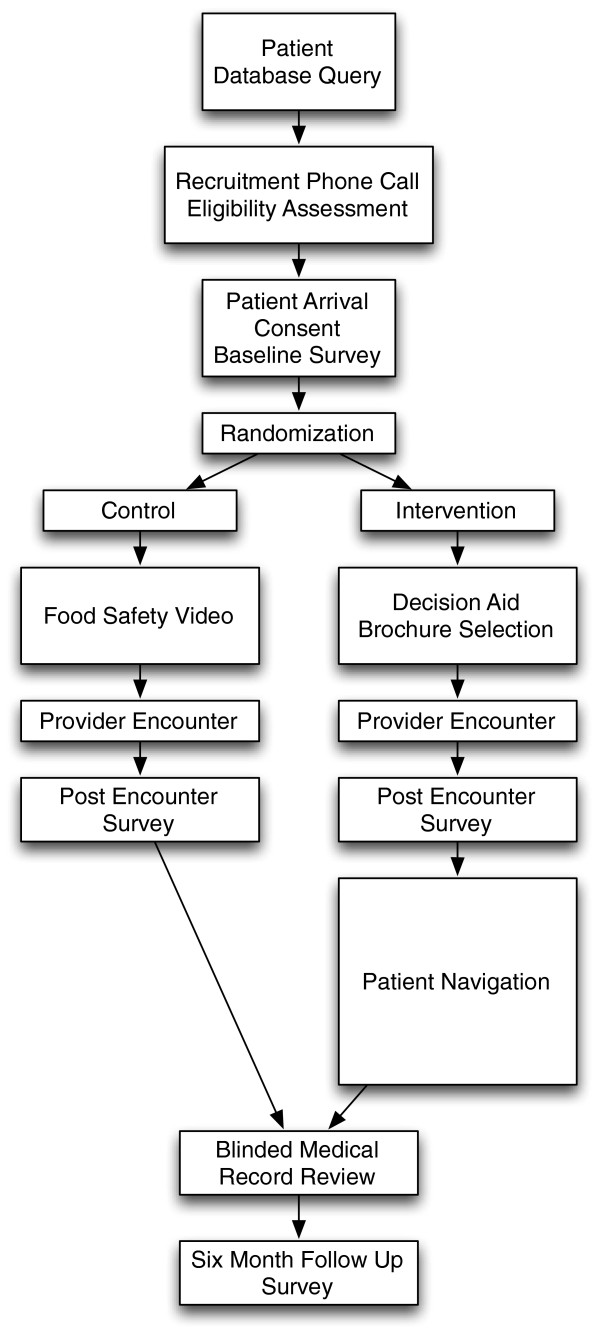
CHOICES/OPCIONES study flow.

Informed by Prochaska’s transtheoretical model and ‘stages of change’ and Bandura’s social cognitive theory [31,32], our conceptual framework (Figure 1) illustrates the steps involved in CRC screening and how we hypothesize that the components of our intervention will affect the process. The decision aid acts mainly to increase patient knowledge, self-efficacy, and intent, and promotes informed decision making regarding choice of screening modality and communication with a healthcare provider. The patient navigator primarily facilitates completion of screening by addressing additional barriers that often supervene even after a screening test has been ordered. However, a patient navigator may also help to build on the knowledge, intent, and self-efficacy established by decision aid viewing.

### Study objectives

The specific objectives of this study are:

1. To determine the effect, relative to usual care, of a practice-based intervention that includes a CRC screening decision aid plus patient navigation on CRC screening completion in diverse, vulnerable primary care patient populations.

2. To determine (a) the intervention effect on knowledge, self-efficacy, intent, and clinical communication about CRC screening and (b) how these factors influence the effectiveness of the intervention on screening test completion.

3. To explore whether patient ethnicity, acculturation, language preference, or health insurance status moderate the relationship between the intervention and screening behavior.

Our main hypotheses are that an intervention including a CRC screening decision aid combined with bilingual and bicultural patient navigators will improve patients’ screening-related knowledge and self-efficacy, promote intention to be screened and patient-provider communication about screening, facilitate test ordering, and result in a significant increase in successful completion of CRC screening.

## Methods/Design

### Overview of study design

As illustrated in Figure 1, the CHOICES/OPCIONES trial is a multisite, two-arm pragmatic randomized controlled trial testing the effect of an intervention, including a CRC screening decision aid and a patient navigator, in primary care clinics serving diverse, vulnerable patients. The combination of two interventions represents a novel approach to promoting cancer screening in vulnerable populations. The primary outcome is CRC screening test completion at six months. Secondary outcomes include CRC screening knowledge, screening self-efficacy, intention to be screened, and patient-provider communication about CRC screening. This study is approved by the Institutional Review Boards at the University of North Carolina (Study # 09–0537), the University of New Mexico (Study ID 12–263), and the Carolinas HealthCare System (File # 12-13-03E).

### Study participants and setting

The trial will recruit approximately 300 participants, including at least 150 Latinos from the two clinic sites over approximately 15 months. Enrolled patients will be aged 50 to 75 years, at average risk of CRC, and not currently undergoing CRC screening according to current US Preventive Services Task Force recommendations [33]. Patients will be excluded if they are unable to speak either English or Spanish, or have severe cognitive, visual, or hearing impairment that would prevent decision aid viewing. Research assistants will review the appointment schedule and medical records to identify potentially eligible participants prior to or on the day of a visit. After determining eligibility, interested participants will complete a signed consent form and provide authorization under the terms of the Health Insurance Portability and Accountability Act.

The two study sites are in Charlotte, North Carolina and Albuquerque, New Mexico, and are parts of larger, non-profit healthcare systems. Both sites serve racially and ethnically diverse, low-income communities. The Charlotte, NC, site is a Community Health Center in northeast Charlotte. The clinic serves approximately 12,000 patients, among whom there is a large and growing Latino population. The clinic site in Albuquerque, NM, is located in the International District of Albuquerque, the most densely populated and ethnically diverse sector of the city. The clinic serves more than 5,000 patients, nearly half of whom are Latino.

### Randomization and blinding

The trial will randomize individual patients, stratified by site, in a 1:1 ratio of intervention to control using randomly permuted blocks with random block sizes. The coordinating site (the University of North Carolina) will produce, for each study site, sequentially numbered, sealed, opaque envelopes containing the randomized arm assignments. The research assistants, who will not know the randomization scheme, will perform the randomization by opening the envelopes sequentially as they enroll study participants. Health care providers at the clinic sites will not be actively notified of patient enrollment in the study. For budgetary reasons, the research assistant conducting the enrollment and index visit data collection will also be the patient navigator, and therefore it is not feasible to blind the research assistant to treatment assignment after randomization occurs. However, a separate, blinded member of the research team will determine the primary study outcome of CRC screening test completion (based on medical record review at six months). In addition, the study biostatistician will program the primary models for addressing each of the aims using dummy treatment assignments and will remain blinded to actual treatment assignments until the models, along with any related assumptions, have been assessed and finalized.

### Intervention and comparison

Participants randomized to the intervention group will receive a combined intervention consisting of CRC screening decision aid and assistance from a trained patient navigator. Participants in the control group will view an attention control video about food safety in their preferred language (English or Spanish) prior to the provider visit and usual care. We chose to use an attention control video so that the structure of the control arm mirrored the intervention arm. The food safety topic was chosen to provide information that is reasonably salient to the control arm participants but that would not be likely to affect conversation during the physician encounter.

#### Decision aid

The decision aid is available in both English and Spanish. The English version was developed and tested rigorously, and has been continually revised and updated to reflect changes in evidence [22,34]. The Spanish version was adapted from the English version using a rigorous cultural and linguistic adaptation and evaluation process [25,35]. The decision aids are 14 to 16 minute long videos and were developed to promote CRC screening, while presenting a balanced view about the choice of screening modality (colonoscopy versus fecal occult blood testing). Participants assigned to the intervention group will view the decision aid prior to the physician visit. Both the English and Spanish versions are designed to be accessible across all literacy levels by using easy-to-understand narration, vignettes, graphics and animations. Patients are introduced to the rationale of CRC screening and the two most widely available screening modalities (colonoscopy and fecal occult blood testing), and the key attributes of both methods are explained, including the frequency of testing, discomfort, costs, risks, and effectiveness. At the end of each decision aid, the viewer is prompted to choose a brochure with a traffic light color scheme that signals his or her readiness to be screened, informed by Prochaska’s transtheoretical model (red, pre-contemplation; yellow, contemplation; green, action) [22,31].

#### Patient navigation

The second component of the intervention is navigation by a trained bilingual and bicultural patient navigator. Navigation will involve both initial face-to-face contact and semistructured phone contact. The role of the patient navigator will be to help patients overcome barriers to completion of CRC screening. The patient navigator will briefly meet the participant after the provider encounter to find out if screening decisions were made and if assistance in carrying out the screening plan is needed. The patient navigator will then attempt to contact intervention participants at two weeks and (at the patient navigator’s discretion) up to four additional times, as necessary, after the clinic visit to assist with screening test completion. Specific counseling will be tailored based on individual patient factors, including choice of test strategy (fecal occult blood testing or colonoscopy), follow-up appointments (including a follow-up colonoscopy for a positive fecal occult blood test), screening barriers, and stage of readiness for screening. For example, a patient who is considering screening (contemplation) might not have received a fecal occult blood testing kit at the index visit, but may later wish to receive the cards by mail. Another patient who is ready for screening (preparing for action) might have had a colonoscopy ordered by the provider, but may be having difficulty understanding how to schedule an appointment, or how to complete the bowel preparation procedure.

### Data collection and measures

#### Overview

Data collection will take place at three time points: baseline, immediately after the physician visit, and at six months after the initial study visit. Measures and timing of measures are shown in Table 1. Patient questionnaires will be administered orally in English or Spanish.

**Table 1:** Time points for principal measures

**Domain**	**Timing**		
	**Baseline**	**Post encounter**	**6 months**
Demographics and covariates	×		
Knowledge about screening	×	×	×
Acculturation	×		
Patient-provider communication		×	
Intention to be screened		×	×
Self-efficacy		×	×
Screening completion			×
Navigation process		×	×
Navigation satisfaction			×

#### Primary outcome

The primary outcome of this study will be completion of a CRC screening test within six months after the initial study visit. Screening test completion will be assessed through a blinded medical record review. Patients will be considered current with screening if there is evidence of completion of a recommended screening test including home fecal occult blood testing, flexible sigmoidoscopy, or colonoscopy. We will record screening modality and results for all tests. For positive fecal occult blood tests, screening will not be considered ‘completed’ unless a follow-up colonoscopy is completed or is pending.

#### Secondary outcomes and other measures

Baseline measures will capture demographics, insurance status, health literacy, language preference, and acculturation (Latinos only). We will also assess CRC knowledge, self-efficacy, and intention to be screened at baseline, using measures from our preliminary studies [25]. Immediately after the provider visit, we will re-assess knowledge, self-efficacy, and intention to be screened, as well as provider-patient communication about CRC screening. At six months, we will re-assess knowledge, self-efficacy, and intention to be screened in the future.

### Analytic approach and power

#### Analyses

We will follow the intention-to-treat principle for all analyses. Our analysis of the primary hypothesis will be a direct, unadjusted comparison of proportions screened in intervention versus control arms using a Mantel-Haenszel *χ*-squared test, controlling for site, conducted at the 0.05 significance level. Latino participants will be examined in a separate subgroup analysis. If substantial differences known to be associated with CRC screening are present at baseline between the intervention and control arms, multiple logistic regression analysis will be used to adjust for these variables. We will, similarly, use logistic regression models to explore whether ethnicity, patient language preference, or health insurance moderate the relationship between the intervention and screening behavior by testing appropriate interaction terms, each at the 0.05 significance level. If any interaction is significant, the intervention will be tested within the respective subgroups using the model with appropriate contrast statements.

**Figure 2:**
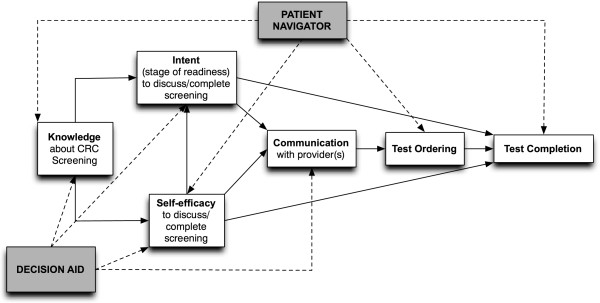
Conceptual model for the CHOICES/OPCIONES project.

To address the second objective, we will apply a structural equation model (specifically, a path analysis with manifest variables) with all variables incorporated appropriately for their scale (that is, knowledge will be ordinal, while self-efficacy, intent, and communication will be categorical). The model will use data from all patients and will control for baseline knowledge, self-efficacy, intent, ethnicity, and site as exogenous variables. The primary model will include indirect effects of the intervention on the outcome through the potential mediating variables along with the direct effect, to allow assessment of whether any mediation is complete or partial. However, in the primary model, no structural model will be imposed relating the mediating variables to one another, beyond simple correlations. As a supportive, secondary, analysis, we will also fit a model that imposes the structural model depicted in Figure 2 and will assess its fit using standard fit indices (that is, root mean squared error of approximation, Bollen’s incremental index, and the Tucker-Lewis index). Nested models will be compared using *χ*-squared difference tests at the 0.05 level.

#### Power calculations

We are planning to recruit at two clinics for 15 to 20 months each. We anticipate being able to enroll about two or three patients per week at each clinic for a total sample ranging from 250 to 380. This will provide more than adequate power to answer the primary research question of intervention effectiveness among all participants and reasonable power to test the effectiveness among Latino participants. If screening status is assessed for at least 90% of enrolled participants, a sample size of 250 will provide at least 90% power to detect a 20% difference in screening completion rate between the groups, assuming equal group sizes, two-sided *α* of 0.05, and a 20% screening rate in the control group. Under these same assumptions, enrolling 150 Latino participants will provide at least 70% power to detect a 20% difference in screening completion rates.

## Discussion

The CHOICES/OPCIONES trial will help fill several gaps in our understanding of how to improve rates of CRC screening among vulnerable populations. First, although several trials have tested either patient navigation or decision aid interventions to increase informed choice and promote CRC screening, this will be the first clinical trial, to our knowledge, to test a combination of the two approaches [36-39]. Second, this trial will test a pragmatic intervention delivered in a real-world clinic-based context. In contrast with studies that deliver patient navigation remotely (that is, only by telephone), this study will use bilingual navigators who are encountered at the clinical site and therefore likely to be viewed as part of the clinical team. Thus, findings may have implications for clinical and payment policies that facilitate incorporation of patient navigation for cancer screening into patient-centered medical home models of care [40].

Third, this trial will advance scientific knowledge regarding methods of overcoming cancer screening disparities for Latino populations, who make up the largest and fastest growing racial or ethnic minority group in the USA and who have substantially lower CRC screening rates than non-Latinos. Fourth, by measuring how viewing a cancer screening decision aid before a primary care visit affects intermediate outcomes (screening-related knowledge, self-efficacy, intent, and communication with a healthcare provider), the study will enhance our mechanistic understanding of how patient-directed interventions affect communication, decision making, and screening behavior. Specifically, the study will help elucidate the causal role that these intermediate factors play in mediating screening behavior. Understanding these causal links is important in designing effective cancer screening behavioral interventions.

Lastly, the exploratory aims of the study have the potential to shed light on how this kind of intervention interacts with key patient-level demographic factors of health insurance and language preference. These findings may inform future research and policy questions, such as those related to healthcare access for immigrant populations, and the degree to which these kinds of intervention should target Latino populations broadly, or should be focused more specifically on the more vulnerable limited English proficiency subpopulations.

## Trial status

This trial is currently enrolling participants. Data collection began in January 2014, and will continue until December 2015.

## Abbreviations

CRC: colorectal cancer.

## Competing interests

The authors declare that they have no competing interests.

## Authors’ contributions

ATB participated in the study design and drafted the manuscript. CMG, MP, RLR, RMH, AM, BUH, MAW, HT, KH, and DR participated in the study design. MAW also conducted the power calculations and designed the analytic approach. KH also provided assistance in the literature review. DR also conceived the study. All authors critically reviewed the manuscript for intellectual content, approved the final version, and have agreed to be accountable for all aspects of the work.
